# The tyrosine kinase inhibitor imatinib mesylate suppresses uric acid crystal-induced acute gouty arthritis in mice

**DOI:** 10.1371/journal.pone.0185704

**Published:** 2017-10-05

**Authors:** Laurent L. Reber, Philipp Starkl, Bianca Balbino, Riccardo Sibilano, Nicolas Gaudenzio, Stephan Rogalla, Steven Sensarn, Dongmin Kang, Harini Raghu, Jeremy Sokolove, William H. Robinson, Christopher H. Contag, Mindy Tsai, Stephen J. Galli

**Affiliations:** 1 Department of Pathology, Stanford University School of Medicine, Stanford, California, United States of America; 2 Sean N. Parker Center for Allergy Research, Stanford University School of Medicine, Stanford, California, United States of America; 3 Department of Immunology, Unit of Antibodies in Therapy and Pathology, Institut Pasteur, Paris, France; 4 Institut National de la Santé et de la Recherche Médicale, Paris, France; 5 Université Pierre et Marie Curie, Paris, France; 6 Departments of Bioengineering, Radiology, and Pediatrics Division of Neonatology, Stanford University School of Medicine, Stanford, California, United States of America; 7 Molecular Imaging Program at Stanford, Stanford, California, United States of America; 8 Department of Life Science, Ewha Womans University, Seoul, Korea; 9 Department of Medicine, Stanford University School of Medicine, Stanford, California, United States of America; 10 Geriatric Research Education and Clinical Center, Veterans Affairs Palo Alto Health Care System, Palo Alto, California, United States of America; 11 Department of Microbiology and Immunology, Stanford University School of Medicine, Stanford, California, United States of America; Universidad de Castilla-La Mancha, SPAIN

## Abstract

Gouty arthritis is caused by the deposition of monosodium urate (MSU) crystals in joints. Despite many treatment options for gout, there is a substantial need for alternative treatments for patients unresponsive to current therapies. Tyrosine kinase inhibitors have demonstrated therapeutic benefit in experimental models of antibody-dependent arthritis and in rheumatoid arthritis in humans, but to date, the potential effects of such inhibitors on gouty arthritis has not been evaluated. Here we demonstrate that treatment with the tyrosine kinase inhibitor imatinib mesylate (imatinib) can suppress inflammation induced by injection of MSU crystals into subcutaneous air pouches or into the ankle joint of wild type mice. Moreover, imatinib treatment also largely abolished the lower levels of inflammation which developed in *IL-1R1*^*-/-*^ or *Kit*^*W-sh/W-sh*^ mice, indicating that this drug can inhibit IL-1-independent pathways, as well as mast cell-independent pathways, contributing to pathology in this model. Imatinib treatment not only prevented ankle swelling and synovial inflammation when administered before MSU crystals but also diminished these features when administrated after the injection of MSU crystals, a therapeutic protocol more closely mimicking the clinical situation in which treatment occurs after the development of an acute gout flare. Finally, we also assessed the efficiency of local intra-articular injections of imatinib-loaded poly(lactic-co-glycolic acid) (PLGA) nanoparticles in this model of acute gout. Treatment with low doses of this long-acting imatinib:PLGA formulation was able to reduce ankle swelling in a therapeutic protocol. Altogether, these results raise the possibility that tyrosine kinase inhibitors might have utility in the treatment of acute gout in humans.

## Introduction

Acute attacks of gout are initiated by the deposition of uric acid and generation of monosodium urate (MSU) crystals in joints. Gout’s prevalence has increased recently, with more than 8 million adults suffering from gout in the US alone [1]. Despite many treatment options for gout, including urate lowering therapy, chronic refractory gout remains a challenging problem, especially in patients with polyarticular and/or tophaceous disease, and can result in functional impairment, joint destruction, and reduced quality of life [2].

Though IL-1 blockade has demonstrated significant utility in prevention of gout flares [3] and in amelioration of symptoms among patients suffering from chronic refractory gout [4], there is still a sizable minority of patients who remain refractory and/or experience breakthrough of gouty inflammation despite these powerful targeted therapies. Notably, the FDA declined approval of the anti-IL-1 agent canakinumab, citing safety concerns as well as unconvincing evidence of the agent’s ability to reduce the frequency of gout flares. However, the FDA panel did agree that there is a serious need for alternative treatment for those patients unresponsive to current therapies [5].

Tyrosine kinase inhibitors such as imatinib mesylate (imatinib) have demonstrated therapeutic benefit in a model of experimental arthritis in mice [6] and in rheumatoid arthritis in humans [7–9]. Imatinib is an FDA-approved drug which targets a spectrum of tyrosine kinases such as Bcr-Abl, KIT and PDGFR [10]. Imatinib is used clinically for the treatment of several diseases including chronic myeloid leukemia (CML) and KIT—positive gastrointestinal stromal tumors (GISTs) [10]. To the best of our knowledge, the effect of imatinib or other tyrosine kinase inhibitors on gout has not yet been assessed.

In this report, we show that the tyrosine kinase inhibitor imatinib mesylate can suppress ankle swelling and synovial inflammation in a mouse model of MSU crystal-induced acute arthritis. Our results raise the possibility that tyrosine kinase inhibitors might have utility in the treatment of acute gout in humans.

## Materials and methods

### Mice

C57BL/6J (WT) mice and *IL-1R1*^*-/-*^ (B6.129S7-*Il1r1*^*tm1Imx*^/J) mice on the C57BL/6 background were purchased from Jackson Laboratories (Bar Harbor, ME) and either bred at the Stanford University Research Animal Facility or used for experiments after maintaining the mice for at least two weeks in our animal facility. C57BL/6-*Kit*^*W-sh/W-sh*^ mice were originally provided by Peter Besmer (Molecular Biology Program, Memorial Sloan-Kettering Cancer Center, New York, NY, USA); we then backcrossed these mice to C57BL/6J mice for more than 11 generations (these mice are available from Jackson Laboratories [B6.Cg-*KitW-sh*/HNihrJaeBsmGlliJ]). We used age-matched male mice for all experiments. All animal care and experimentation were conducted in compliance with the guidelines of the National Institutes of Health and with the specific approval of the Institutional Animal Care and Use Committees of Stanford University and of the Animal Ethics committee CETEA (Institut Pasteur, Paris, France) registered under #C2EA-89.

### Preparation and intra-articular injection of MSU crystals

MSU crystals were prepared as described previously [11, 12]. 1 g of uric acid (Sigma) in 180 mL of 0.01 M NaOH was heated to 70°C. NaOH was added as required to maintain pH between 7.1 and 7.2 and the solution was filtered and incubated at room temperature with slow and continuous stirring for 24 h. MSU crystals were kept sterile, washed with ethanol, dried, autoclaved and re-suspended in PBS by sonication. MSU crystals contained < 0.005 EU/mL endotoxin (LAL endotoxin assay, GenScript). We injected 0.5 mg MSU (or 2.0 mg MSU in *IL-1R1*^*-/-*^ mice) intra-articularly (i.a.) in 10 μL PBS into one ankle joint and PBS alone into the contra-lateral ankle. We used Microliter Syringes #705 (Hamilton) with 27G needles for all i.a. injections (except for injections of 2.0 mg MSU in *IL-1R1*^*-/-*^ mice, which were performed using 25G needles). Injections were performed under isoflurane anesthesia, and the quality of i.a. injection was controlled by assessing the location of MSU crystal deposition, via histology, using ankle tissue collected 24 h after the injection. Ankle swelling was measured at different time points using a precision caliper (Fisherbrand Traceable Digital Calipers; Fischer Scientific). To avoid any attribution bias, ankle swelling was measured by an investigator who was blinded to the group allocation. Mice were euthanized by CO_2_ inhalation 24 h after injection of MSU crystals.

### Treatment with imatinib mesylate

Imatinib mesylate (LC Laboratories) suspended in PBS was administered intra-peritoneally (i.p.) (30 or 100 mg/kg in 200 μL PBS) at 24, 15 and 1 h before and 6 h after (prophylactic model) or 1 h and 6 h after (therapeutic model) i.a. injection of MSU crystals. Only PBS was injected i.p. into control mice. In some experiments, imatinib mesylate (obtained from Sigma) was suspended in PBS and administered by gavage at 100 mg/kg in a total volume of 100 μl twice a day starting one day before i.a. injections of MSU crystals.

### Preparation and injection of imatinib-loaded PLGA nanoparticles

Imatinib-loaded PLGA particles were prepared at the Biomaterials and Advanced Drug Delivery (BioADD) facility (Stanford, USA). Imatinib (0.1 g) was dissolved in 20 mL of acetone with sonication for 30 min. Poly(lactic-co-glycolic acid) PLGA (0.9 mg) was dissolved in 180 mL acetone and mixed with imatinib solution. The solution mixture was spray-dried using Buchi-290 mini spray dryer. The set parameters were: inlet temperature: 40°C; outlet temperature: 30°C; aspirator at 90%; solution feed pump at 5% and nitrogen flow at 40 mm. The feeding solution container and spray dryer compartments were protected from light during the process. The dry powder was collected and stored at 4°C. After spray drying, the imatinib-loading efficiency in the PLGA particles was 11.6%, as determined using the LCMS method (4000 QTRAP HPLC-MS/MS System [AB SCIEX] with Shimadzu Prominence LC system, equipped with system controller CBM-20A, Binary LC-20AD pump, and SIL-20AC autosampler). Vehicle-loaded PLGA nanoparticles were prepared using the same conditions but without addition of imatinib, and were used as controls. Vehicle-loaded and imatinib-loaded PLGA nanoparticles were suspended in PBS at 0.5 mg/ml. 20 μl of imatinib-loaded PLGA nanoparticles suspension were injected intra-articularly (i.a.) in one ankle and 20 μl of vehicle-loaded PLGA nanoparticles suspension were injected i.a. into the contra-lateral ankle 1 h and 6 h after (therapeutic model) i.a. injection of MSU crystals in both ankles.

### Air pouch model

Mice were anesthetized with isofluorane, the back skin was shaved, and 3 ml of sterile air was injected subcutaneously (s.c.) into the back to form an air pouch. Three days after the first injection, an additional 3 ml of sterile air were re-injected into the pouch. Three days later, we injected 3 mg of MSU crystals in 1 ml PBS, or 1 ml of PBS alone, into the air pouches. Six hours after injection of MSU crystals or vehicle, mice were euthanized by CO_2_ inhalation, and exudates were collected and centrifuged at 500 *g* for 5 min at room temperature. Cells were counted with a hemocytometer. Characterization of leukocyte subpopulations was performed by flow cytometry. Concentrations of IL-1β in air pouch exudates were analyzed by ELISA (eBioscience).

### Flow cytometry

We used flow cytometry to identify neutrophils (Ly6G^+^; CD11b^+^), monocytes (Ly6G^-^; CD11b^+^), and eosinophils (SSC^high^; Siglec-F^+^), in air pouch exudates. Red blood cells were lysed by treatment with pH 7.3 ACK lysis buffer (0.15 M NH_4_Cl, 1 mM KHCO_3_, and 0.1 mM EDTA, pH 8.0) 2 times for 5 min each. Cells were blocked with unconjugated anti—CD16/CD32 antibodies (BioXcell) on ice for 5 min and then stained with APC/Cy7 anti-Ly6G (1A8, BD Biosciences), PE anti-Siglec-F (E50-2440, BD Biosciences) and eFluor450 anti-CD11b (M1/70, eBioscience) antibodies on ice for 30 min. Data were acquired using a FACSCanto II flow cytometer (BD Biosciences). Data were analyzed with FlowJo 9.5.3. software (TreeStar). Dead cells (identified by staining with LIVE/DEAD aqua amine; Invitrogen) were not included in the analysis.

### Histologic analysis

Joints were fixed in 10% formalin, decalcified for 10 days in EDTA 0.5 M pH = 8, embedded in paraffin, and 4-μm sections were stained with hematoxylin & eosin (H&E) for histologic examination of leukocytes. Images were captured with a Nikon (Belmont, CA) E1000M microscope using a Spotflex camera and Spot version 5.1 software (Diagnostic Instruments, Sterling Heights, MI).

### Imaging analysis of MPO activity

We assessed MPO activity in the ankle joint 24 h after i.a. injection of MSU crystals using *in vivo* bioluminescence imaging [13]. Luminol was injected, i.p., (Sigma; 200 mg/kg in 200 μL PBS) into the mice and the animals were anesthetized (isoflurane inhalation), and imaged in an IVIS 200 or IVIS Spectrum instrument (Perkin-Elmer) with a 5 min acquisition time for the bioluminescent image using an open filter and beginning 5 min after injection of luminol. Acquisition and analysis of data were performed using the LivingImage software (Perkin-Elmer).

### Statistical analyses

Data are presented as means + SEM or means ± SEM. Differences between groups were assessed for statistical significance by ANOVA or by using an unpaired Mann-Whitney *U* test or an unpaired Student *t* test, as indicated in the legends. As indicated in each figure legend, all *in vivo* experiments were done 2 or 3 independent times, and the “*n*” in each legend refers to the total number of mice used for that experiment, after pooling the results of the replicate experiments done. *P* values < 0.05 were considered statistically significant.

## Results

### Intra-peritoneal treatment with imatinib reduces MSU crystal-induced inflammation in the air-pouch model

**Fig 1:**
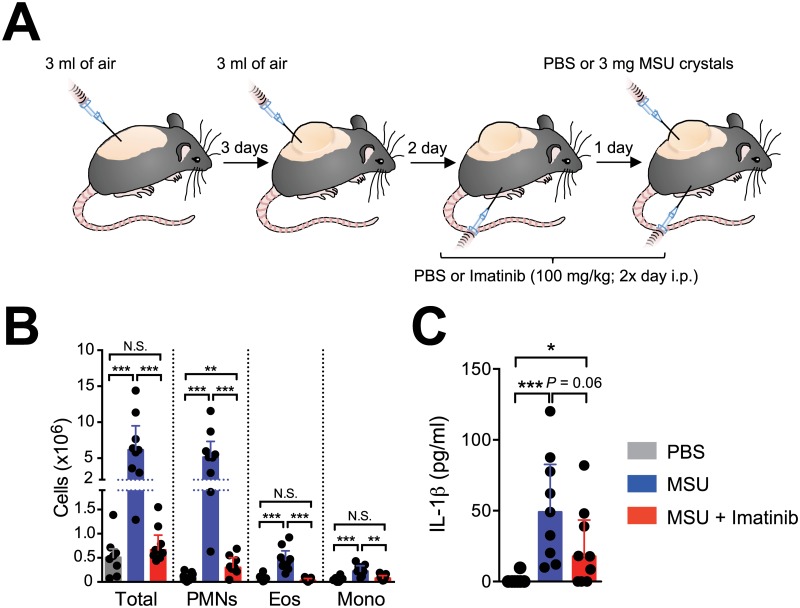
Prophylactic treatment with imatinib reduces MSU crystal-induced inflammation in the air pouch model. (**A**) Experimental outline. Mice were anesthetized, shaved on the back, and injected with 3 ml of sterile air subcutaneously (s.c.) into the back to form an air pouch. Three days after the first injection, an additional 3 ml of sterile air were injected into the pouch. Three days later, we injected 3 mg of MSU crystals in 1 ml PBS, or PBS alone, into the air pouches. Mice received intra-peritoneal (i.p.) injections of imatinib (100 mg/kg i.p. in 200 μl PBS) or vehicle (PBS) twice a day starting 24 h before the injection of MSU crystals. (**B**) Number of total cells, neutrophils (Ly6G^+^; CD11b^+^), eosinophils (SSC^high^; Siglec-F^+^) and monocytes (Ly6G^-^; CD11b^+^) in air pouch exudates collected 6 h after injection of MSU crystals or PBS. (**C**) Levels of IL-1β in air pouch exudates collected 6 h after injection of MSU crystals or PBS. Data in **B** and **C** are depicted as values from individual mice, with histograms indicating medians + quartiles of data pooled from the three independent experiments performed with a total of *n* = 8 (‘PBS’ group) or *n* = 9 (‘MSU’ and ‘MSU + Imatinib’ groups) mice. *, ** or *** = *P* < 0.05, 0.01 or 0.001 *vs*. indicated group by Mann-Whitney *U* test.

We first assessed the effect of pretreatment of mice with imatinib 100 mg/kg twice per day intraperitoneally (i.p.) on inflammation induced by MSU crystal injection in preformed subcutaneous air pouches [14]. Injection of MSU crystals into air pouches induced an increase in numbers of total cells, neutrophils, eosinophils and monocytes at 6 h, as compared to PBS injection (Fig 1A and 1B). Numbers of all these cell populations were highly reduced in mice treated with imatinib (Fig 1A and 1B). Since MSU crystal-induced inflammation critically depends on IL-1β [12], we also measured concentrations of this inflammatory cytokine in air pouch lavages collected 6 h after injection of MSU crystals. Injection of MSU crystals induced an increase in concentrations of IL-1β as compared to PBS injection. While the data show a trend in the ability of imatinib to reduce IL-1β concentrations in this model, the difference between the vehicle- and imatinib-treated groups did not reach statistical significance (*P* = 0.06) (Fig 1C).

### Intra-peritoneal treatment with imatinib diminishes MSU crystal-induced acute arthritis in mice

**Fig 2:**
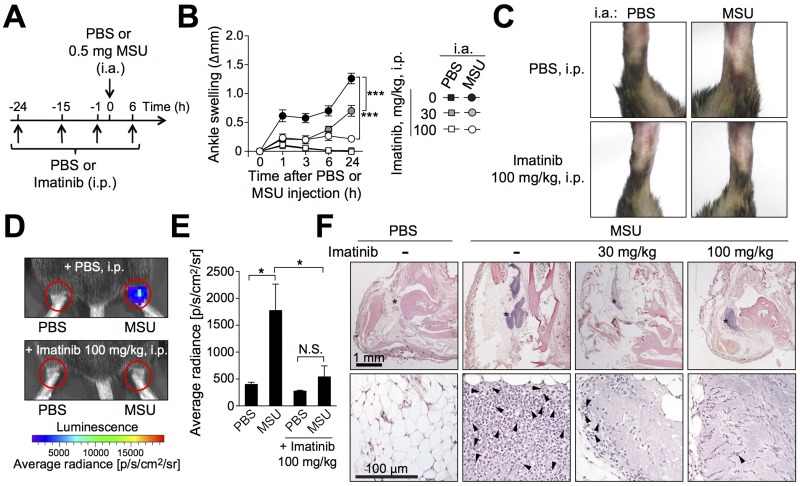
Prophylactic treatment with imatinib reduces MSU crystal-induced ankle inflammation. (**A**) C57BL/6J mice received intra-peritoneal (i.p.) injections of imatinib (30 or 100 mg/kg i.p. in 200 μl PBS) or vehicle (PBS) twice a day starting 24 h before intra-articular (i.a.) injection of MSU crystals (0.5 mg in 10 μL) in one ankle and vehicle (10 μl PBS) in the contra-lateral ankle. (**B** and **C**) Time course (**B**) and representative photographs (**C**) (at 24 h) of MSU crystal-induced ankle swelling in mice pre-treated with imatinib or PBS (*n* = 12-15/group). Data in **B** are shown as means ± SEM of data pooled from the three independent experiments performed. *** = *P* < 0.001 *vs*. indicated group by repeated measures two-way ANOVA. (**D** and **E**) Image of photon emission, pseudocolor, superimposed over grayscale reference image (**D**) and quantification (**E**) of myeloperoxidase (MPO)-induced bioluminescence in the ankle joint 24 h after i.a. injections of MSU crystals or PBS in mice pre-treated with imatinib or PBS (*n* = 10/group). Bioluminescence intensity was calculated in defined regions of interest (ROI) around the ankle joints (red circles in **D**). Data are shown as means ± SEM of data pooled from the four independent experiments performed. * = *P* < 0.05 *vs*. indicated group; N.S.: not significant (*P* > 0.05) by unpaired Student *t* test. (**F**) H&E-stained sections of ankles 24 h after injection of 0.5 mg MSU crystals in mice pre-treated with imatinib or PBS. Lower panels in **F** are enlargement of the areas marked (*) in the upper panels, with solid arrowheads depicting a few of the many neutrophils present at sites of MSU crystal injection, especially in the section from the mouse not treated with imatinib. Original magnification: x1 for upper panels, x20 for lower panels.

We next tested the possible effects of the tyrosine kinase inhibitor imatinib in a mouse model of acute gout consisting on performing intra-articular injections of MSU crystals into the ankle joint [11]. We found that imatinib can diminish ankle swelling in a dose-dependent manner in this model when administered i.p. twice a day starting one day before MSU crystal injection (Fig 2A–2C). Mice treated i.p. with 100 mg/kg imatinib still developed small but significant ankle swelling 24 h after injection of MSU crystals. Such swelling completely resolved by 72 h, suggesting that treatment with imatinib does not delay the resolution of the inflammatory process in this model (S1 Fig). Since injection of MSU crystals leads to the marked recruitment of neutrophils in mice [11, 12], and high levels of neutrophils are found in synovial fluid from gout patients [15], we also assessed whether imatinib influenced neutrophil activity in the ankle joint in this acute gout model. We used a recently described non-invasive *in vivo* bioluminescence imaging method to quantify the activity of myeloperoxidase (MPO), an enzyme mainly produced by neutrophils. This method allows quantification of bioluminescence emitted from the oxidation of exogenously administered luminol by active MPO [13]. We detected significant elevation of the bioluminescent signal in ankle joints that had been injected 24 h earlier with MSU crystals as compared to contra-lateral ankles injected with PBS alone. This MSU crystal-induced elevation of bioluminescence was abrogated by treatment with imatinib (Fig 2D and 2E). Crystal deposits could not be observed directly by histology in our study, since formalin used in the fixation process dissolved the crystals, leaving areas of ‘amorphous material’ [16]. Such areas of amorphous material were present in all groups of MSU crystal-injected mice at 24 h, suggesting that imatinib does not reduce MSU crystal deposits. However, histological examination revealed that the number of leukocytes within and around these areas was markedly reduced in mice treated with imatinib (Fig 2F). Taken together, our results indicated that prophylactic treatment with imatinib can suppress both ankle swelling and accumulation of leukocytes in this mouse model of acute gout.

### Imatinib suppresses IL-1-independent MSU crystal-induced acute arthritis

**Fig 3:**
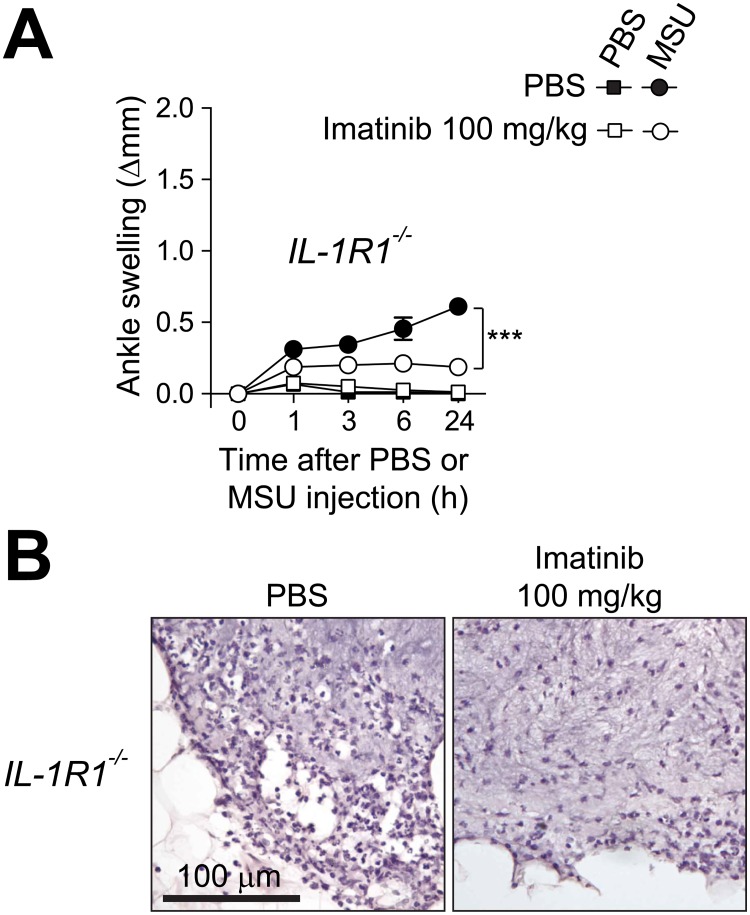
Intraperitoneal treatment with imatinib suppresses IL-1-independent MSU crystal-induced acute arthritis. (**A**) Changes in ankle thickness after i.a injection of 2.0 mg MSU or PBS in *IL-1R1*^*-/-*^ mice (*n* = 8-9/group) pre-treated with imatinib (100 mg/kg) or PBS twice a day starting 24 h before the injection of MSU crystals. Data are shown as means ± SEM of data pooled from the two independent experiments performed. *** = *P* < 0.001 *vs*. indicated group by repeated measures two-way ANOVA. (**B**) H&E-stained sections of ankle showing MSU injection sites 24 h after injection of 2 mg MSU crystals in *IL-1R1*^*-/-*^ pre-treated with imatinib (100 mg/kg) or PBS. Original magnification: x20.

MSU crystals can activate the NLRP3 inflammasome, leading to release of IL-1β [12] and mice deficient for the IL-1 receptor IL-1R1 develop diminished levels of MSU crystal-induced ankle swelling [11]. We here report that *IL-1R1*^*-/-*^ mice can develop significant ankle swelling and leukocyte infiltration when injected with a high dose of MSU crystals (2 mg) (Fig 3A and 3B). Imatinib suppressed such MSU crystal-induced ankle swelling and leukocyte infiltration in *IL-1R1*^-/-^ mice, indicating that the drug can inhibit IL-1-independent pathways contributing to arthritis in this acute gout model (Fig 3A and 3B).

### Imatinib suppresses mast cell-independent MSU crystal-induced acute arthritis

**Fig 4:**
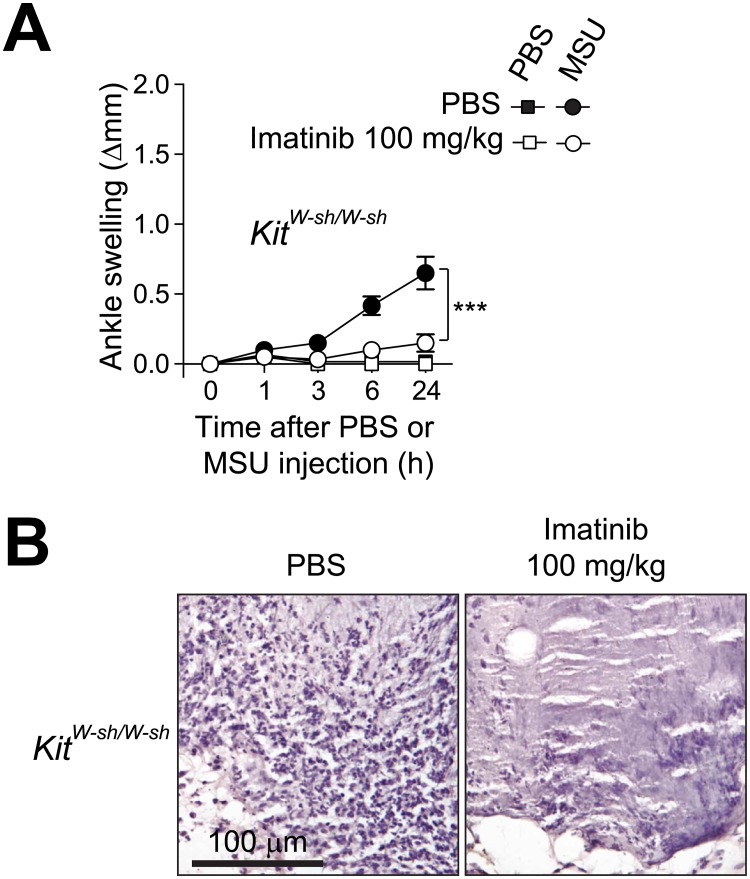
Intraperitoneal treatment with imatinib suppresses mast cell-independent MSU crystal-induced acute arthritis. (**A**) Changes in ankle thickness after i.a injection of 0.5 mg MSU in mast cell-deficient *Kit*^*W-sh/W-sh*^ mice (*n* = 6/group) pre-treated with imatinib (100 mg/kg) or PBS twice a day starting 24 h before the injection of MSU crystals. Data are shown as means ± SEM of data pooled from the two independent experiments performed. *** = *P* < 0.001 *vs*. indicated group by repeated measures two-way ANOVA. (**B**) H&E-stained sections of ankle showing MSU injection sites 24 h after injection of 0.5 mg MSU crystals in *Kit*^*W-sh/W-sh*^ mice pre-treated with imatinib (100 mg/kg) or PBS. Original magnification: x20.

Imatinib can suppress mast cell activation via its effects on KIT [17]. We recently showed that mast cell-deficient mice develop diminished levels of ankle swelling in this acute gout model [11]. We show in the current study that imatinib virtually eliminated the development of the residual MSU crystal-induced ankle swelling and leukocyte infiltration observed in genetically mast cell-deficient *Kit*^*W-sh/W-sh*^ mice, implying that the drug suppressed these features independently of actions on mast cells (Fig 4A and 4B).

### Intra-peritoneal treatment with imatinib suppresses MSU crystal-induced acute arthritis in a therapeutic protocol

**Fig 5:**
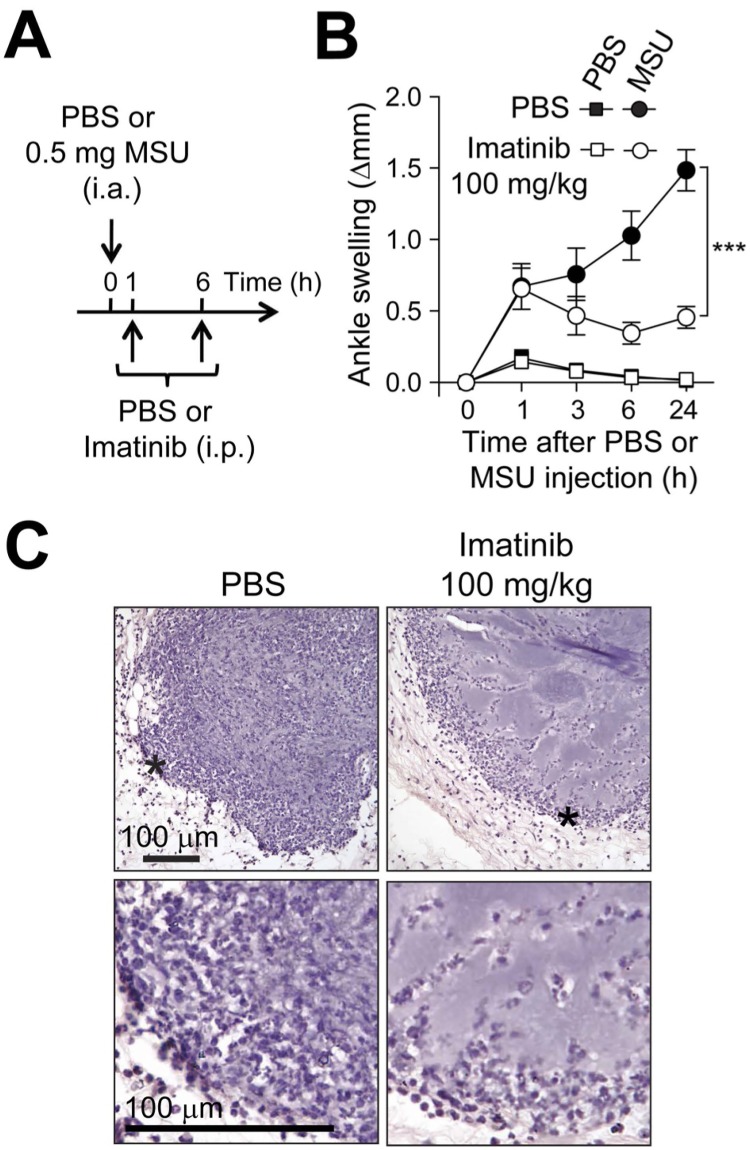
Intraperitoneal treatment with imatinib reduces MSU crystal-induced ankle inflammation in a therapeutic protocol. (**A**) C57BL/6J mice received imatinib (100 mg/kg, i.p.) or vehicle (PBS) 1 and 6 h after i.a. injection of MSU crystals (0.5 mg in 10 μl) in one ankle and vehicle (10 μl PBS) in the contra-lateral ankle. (**B**) Time course of MSU crystal-induced ankle swelling in this therapeutic protocol (*n* = 8-9/group). Data are shown as means ± SEM of data pooled from the three independent experiments performed. *** = *P* < 0.001 *vs*. indicated group by repeated measures two-way ANOVA. (**C**) H&E-stained sections of ankle showing MSU injection sites 24 h after injection of 0.5 mg MSU crystals in the therapeutic protocol. Lower panels in **C** are enlargement of the areas marked (*) in the upper panels. Original magnification: x20.

In order to assess the potential benefit of treating acute gout attacks with imatinib, we administered the drug 1 and 6 h after injection of MSU crystals (Fig 5A). This treatment protocol more closely mimics the clinical situation in which treatment occurs after the clinical development of an acute gout flare. We found that imatinib still was able to reduce MSU crystal-induced ankle swelling and leukocyte infiltration significantly in this therapeutic protocol (Fig 5B and 5C).

### Oral administration of imatinib imatinib reduces MSU crystal-induced acute arthritis

**Fig 6:**
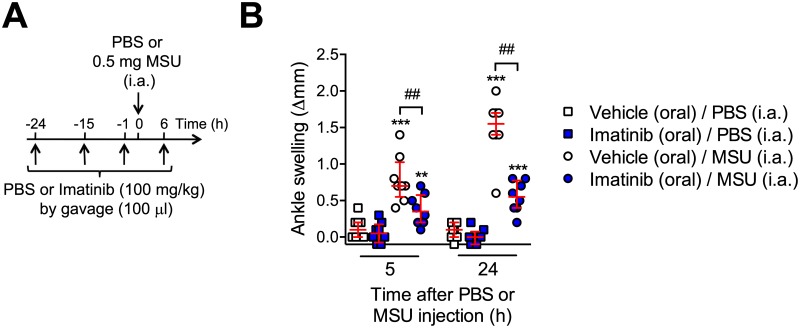
Oral treatment with imatinib reduces MSU crystal-induced acute arthritis. (**A**) C57BL/6J mice received imatinib (100 mg/kg in 100 μl) or vehicle (100 μl PBS) by gavage twice a day starting 24 h before the i.a. injection of MSU crystals (0.5 mg in 10 μl) in one ankle and vehicle (10 μl PBS) in the contra-lateral ankle. (**B**) Ankle swelling 5 h and 24 h after i.a. injection of MSU crystals or vehicle (PBS). Data are depicted as values from individual mice, with histograms indicating medians + quartiles pooled of data pooled from the two independent experiments performed with a total of *n* = 8 mice/group. ** or *** = *P* < 0.01 or 0.001 *vs*. corresponding PBS-treated group, and ^##^ = *P* < 0.01 *vs*. indicated group by Mann-Whitney *U* test.

Because imatinib is given orally in humans, we also assessed the effectiveness of oral treatment with imatinib in our model of acute gout. We administered imatinib orally at 100 mg/kg twice a day starting one day before MSU crystal injection (Fig 6A), a dose that results in a pharmacokinetic profile similar to that in humans on a mid-range dose of 400 mg once daily [18, 19]. This dose of imatinib was sufficient to significantly reduce ankle swelling in our model of acute gout (Fig 6B).

### Intra-articular treatment with imatinib-loaded PLGA nanoparticules reduces MSU crystal-induced acute arthritis in a therapeutic protocol

**Fig 7:**
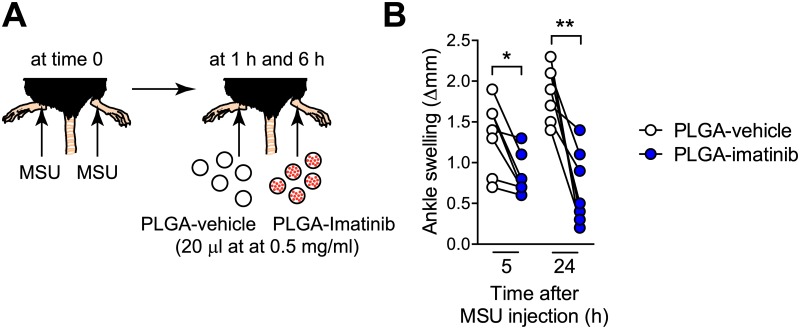
Intra-articular treatment with imatinib-loaded PLGA nanoparticles reduces MSU crystal-induced acute arthritis in a therapeutic protocol. (**A**) C57BL/6J mice were injected i.a. with MSU crystals (0.5 mg in 10 μl) in both ankles at time 0. One hour and 6 hours after MSU crystal injection, mice were injected i.a. with imatinib-loaded PLGA nanoparticles (PLGA-imatinib; 20 μl at 0.5 mg/ml) in one ankle and control nanoparticles (PLGA-vehicle; 20 μl at 0.5 mg/ml) in the contra-lateral ankle. (**B**) Ankle swelling 5 h and 24 h after i.a. injection of MSU crystals (*n* = 7/group). Data are depicted as values from individual mice pooled from the two independent experiments performed. * or ** = *P* < 0.05 or 0.01 *vs*. indicated group using a paired Student’s *t* test.

Finally, in order to reduce risks of side effects, which could be observed with chronic treatments with high doses imatinib in humans, we assessed whether local intra-articular administration of low dose imatinib would be sufficient to reduce ankle swelling in a therapeutic protocol in our model of gout (Fig 7A). In order to reduce the dose of imatinib, we generated a long-acting formulation of imatinib by encapsulating the drug into biodegradable poly(lactic-co-glycolic acid) (PLGA) nanoparticles [20, 21]. We injected imatinib-loaded PLGA nanoparticles i.a. in one ankle and vehicle-loaded PLGA nanoparticles i.a. in the contra-lateral ankle 1 h and 6 h after i.a. MSU crystal injection in both ankles (Fig 7A). We found significantly reduced ankle swelling at both 5 and 24 h after MSU crystal injection in ankles treated with imatinib-loaded PLGA nanoparticles as compared to the contra-lateral ankles treated with vehicle-loaded nanoparticles (Fig 7B).

## Discussion

In this study, we demonstrated that the tyrosine kinase inhibitor imatinib can suppress MSU crystal-induced inflammation in an air-pouch model and in a model of acute gout. Oral or intra-peritoneal treatment of mice with imatinib largely prevented both ankle swelling and leukocyte recruitment in these models. Imatinib remained effective at inhibiting these features in a therapeutic protocol, in which the drug was given after injection of MSU crystals, at a time where significant ankle swelling had already developed. Importantly, local injections of a long acting formulation of the drug consisting of low doses imatinib encapsulated into biodegradable PLGA nanoparticles was sufficient to reduce ankle swelling in a therapeutic protocol.

Treatment with imatinib reduces pathology in mouse models of autoimmune arthritis [6] and was associated with improvement of rheumatoid arthritis symptoms in case reports in humans [7]. The mechanism of action of imatinib in these settings remains largely unknown, but multiple lines of evidence indicate that the drug can inhibit diverse cellular responses that play critical roles in driving arthritis, including inhibition of mast cell activation (via its effects on KIT) [6, 8]. We recently showed that mast cells and mast cell-derived IL-1β are important contributors to MSU crystal-induced ankle swelling in mice [11]. Therefore, it is possible that inhibition of mast cell activation contributes to the anti-inflammatory effects of imatinib in this acute gout model. Treatment with imatinib had no apparent effects on the number or distribution of mast cells in synovial tissue 24 h after injection of MSU crystals (data not shown). Moreover, we found that imatinib can suppress the residual arthritis which develops in genetically mast cell-deficient *Kit*^*W-sh/W-sh*^ mice, indicating that this drug is able to block mast cell-independent pathways of MSU crystal-induced arthritis.

MSU crystal-induced inflammation depends on IL-1β [3, 4, 11, 12, 15]. However, we reported that mice deficient for the IL-1 receptor IL-1R1 develop reduced but still significant ankle swelling as compared to WT mice in this acute gout model [11]. Notably, *IL-1R1*^*-/-*^ mice developed substantial infiltration of leukocytes in the ankle synovium by 24 h after injection of MSU crystals, indicating that IL-1-independent pathways also contribute to such MSU-induced inflammation [11]. We confirm here that *IL-1R1*^*-/-*^ mice develop significant ankle swelling when injected with a high dose of MSU crystals (2 mg). Importantly, treatment of *IL-1R1*^*-/-*^ mice with imatinib suppressed both the ankle swelling and the leukocyte infiltration associated with exposure to this high concentration of crystals, indicating that the drug can inhibit IL-1-independent pathways contributing to arthritis in this acute gout model.

Chronic oral treatment with high doses imatinib can cause a broad range of side effects (reviewed in [22]). We thus tested the efficiency of local treatment of acute gout attacks using a long-term formulation of the drug consisting of low doses imatinib encapsulated into biodegradable PLGA nanoparticles. Loading of a drug into PLGA nanoparticles not only can extend the half-life of the drug *in vivo*, but also enables slow release of the drug over an extended period of time [23]. Such an approach has been used successfully to administer encapsulated proteins and drugs in models of collagen-induced arthritis and antibody-induced arthritis [20, 21]. We show here that imatinib-loaded nanoparticles administered intra-articularly after MSU crystal injection can significantly reduce ankle swelling in our model of acute gout. Ankle swelling was still substantial in the contra-lateral ankle joint injected with vehicle-loaded nanoparticles. Our data thus suggest that imatinib can achieve its anti-inflammatory effects locally in this gout model, at doses that are not likely to induce strong side effects when given in an acute setting.

In summary, our findings indicate that systemic or local treatment with the tyrosine kinase inhibitor imatinib can suppress acute gouty arthritis in mice. Although care should be taken in extrapolating results obtained in mice to humans, our findings raise the possibility that tyrosine kinase inhibitors such as imatinib might have utility in the treatment of acute gout flares in humans.

## Supporting information

S1 Fig Effects of intraperitoneal treatment with imatinib on MSU crystal-induced acute arthritis over 96 h. C57BL/6J mice received intra-peritoneal (i.p.) injections of imatinib (100 mg/kg i.p. in 200 μl PBS) or vehicle (200 μl PBS) twice a day starting 24 h before intra-articular (i.a.) injection of MSU crystals (0.5 mg in 10 μL) in one ankle and vehicle (10 μl PBS) in the contra-lateral ankle. Ankle swelling was measured at the indicated time points up to 96 h after i.a. injection of vehicle or MSU crystals. Data are shown as means ± SEM from n = 6 mice/group. *** = P < 0.001 *vs*. indicated group by repeated measures two-way ANOVA.
(PDF)

S2 Fig *In vitro* analysis of imatinib-loaded PLGA nanoparticles. **A**. Microscopic picture of vehicle-loaded and imatinib-PLGA nanoparticles, showing that the diameter of the particles was up to 5 μm with an average size < 2 μm. **B**. Release of drug from imatinib-loaded PLGA nanoparticle incubated in human synovial fluid (‘normal human synovial fluid’ obtained from BioreclamationIVT, USA). 10 mg of imatinib-encapsulated PLGA particles was suspended in 1 mL of synovial fluid in a 1.5 mL microcentrifuge tube. The suspension was incubated at 37°C on an orbital shaker at 120 rpm. At the indicated time points, the particles in the suspension were spun down for 30 seconds using a mini centrifuge (VWR C1413 Galaxy mini centrifuge), and an aliquot of 100 μL was withdrawn for the analysis. The volume was replenished with 100 μL of fresh synovial fluid. The incubation was continued after resuspending the particles. The experiment was performed in triplicate and the results are expressed as the mean ± SD. The released drug was quantified using a 4000 QTRAP HPLC-MS/MS System (AB SCIEX) with Shimadzu Prominence LC system, equipped with system controller CBM-20A, Binary LC-20AD pump, and SIL-20AC autosampler. HPLC was performed using the following specifications: mobile phase: 90% methanol/10% water/0.1% formic acid, column: Dionex, Acclaim 120 C8, 5 μm, 50 x 2.1 mm, flow rate: 0.3 ml/min, and injection volume: 10 μL. The HPLC was directly coupled to an AB SCIEX 4000 QTRAP triple quadrupole mass spectrometer with electrospray ionization. The mass spectrometer was operated in positive multiple reaction monitoring (MRM) mode, ion source: turbo spray, resolution: Q1 unit; Q3 unit.
(PDF)

S1 File ARRIVE guidelines checklist. (PDF)
