# Supplementary material for: The tyrosine kinase inhibitor imatinib mesylate suppresses uric acid crystal-induced acute gouty arthritis in mice
Source: PLoS One. 2017 Oct 5;12(10):e0185704. doi: 10.1371/journal.pone.0185704 (PMC5628843; doi:10.1371/journal.pone.0185704)
Supplement: S1 Fig — C57BL/6J mice received intra-peritoneal (i.p.) injections of imatinib (100 mg/kg i.p. in 200 μl PBS) or vehicle (200 μl PBS) twice a day starting 24 h before intra-articular (i.a.) injection of MSU crystals (0.5 mg in 10 μL) in one ankle and vehicle (10 μl PBS) in the contra-lateral ankle. Ankle swelling was measured at the indicated time points up to 96 h after i.a. injection of vehicle or MSU crystals. Data are shown as means ± SEM from n = 6 mice/group. *** = P < 0.001 vs. indicated group by repeated measures two-way ANOVA. (PDF) [file pone.0185704.s001.pdf]

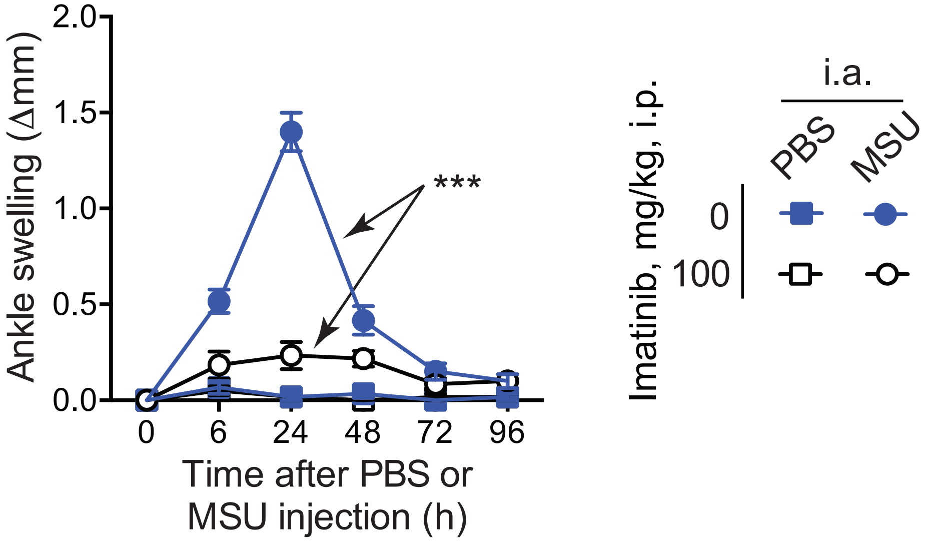

Supplementary Figure S1. Effects of intraperitoneal treatment with imatinib on MSU crystal-induced acute arthritis over 96 h. C57BL/6J mice received intra-peritoneal (i.p.) injections of imatinib (100 mg/kg i.p. in 200 μl PBS) or vehicle (200 μl PBS) twice a day starting 24 h before intra-articular (i.a.) injection of MSU crystals (0.5 mg in 10 μL) in one ankle and vehicle (10 μl PBS) in the contra-lateral ankle. Ankle swelling was measured at the indicated time points up to 96 h after i.a. injection of vehicle or MSU crystals. Data are shown as means ± SEM from n=6 mice/group. *** = P < 0.001 vs. indicated group by repeated measures two-way ANOVA.
