# Supplementary material for: The tyrosine kinase inhibitor imatinib mesylate suppresses uric acid crystal-induced acute gouty arthritis in mice
Source: PLoS One. 2017 Oct 5;12(10):e0185704. doi: 10.1371/journal.pone.0185704 (PMC5628843; doi:10.1371/journal.pone.0185704)
Supplement: S2 Fig — A. Microscopic picture of vehicle-loaded and imatinib-PLGA nanoparticles, showing that the diameter of the particles was up to 5 μm with an average size < 2 μm. B. Release of drug from imatinib-loaded PLGA nanoparticle incubated in human synovial fluid (‘normal human synovial fluid’ obtained from BioreclamationIVT, USA). 10 mg of imatinib-encapsulated PLGA particles was suspended in 1 mL of synovial fluid in a 1.5 mL microcentrifuge tube. The suspension was incubated at 37°C on an orbital shaker at 120 rpm. At the indicated time points, the particles in the suspension were spun down for 30 seconds using a mini centrifuge (VWR C1413 Galaxy mini centrifuge), and an aliquot of 100 μL was withdrawn for the analysis. The volume was replenished with 100 μL of fresh synovial fluid. The incubation was continued after resuspending the particles. The experiment was performed in triplicate and the results are expressed as the mean ± SD. The released drug was quantified using a 4000 QTRAP HPLC-MS/MS System (AB SCIEX) with Shimadzu Prominence LC system, equipped with system controller CBM-20A, Binary LC-20AD pump, and SIL-20AC autosampler. HPLC was performed using the following specifications: mobile phase: 90% methanol/10% water/0.1% formic acid, column: Dionex, Acclaim 120 C8, 5 μm, 50 x 2.1 mm, flow rate: 0.3 ml/min, and injection volume: 10 μL. The HPLC was directly coupled to an AB SCIEX 4000 QTRAP triple quadrupole mass spectrometer with electrospray ionization. The mass spectrometer was operated in positive multiple reaction monitoring (MRM) mode, ion source: turbo spray, resolution: Q1 unit; Q3 unit. (PDF) [file pone.0185704.s002.pdf]

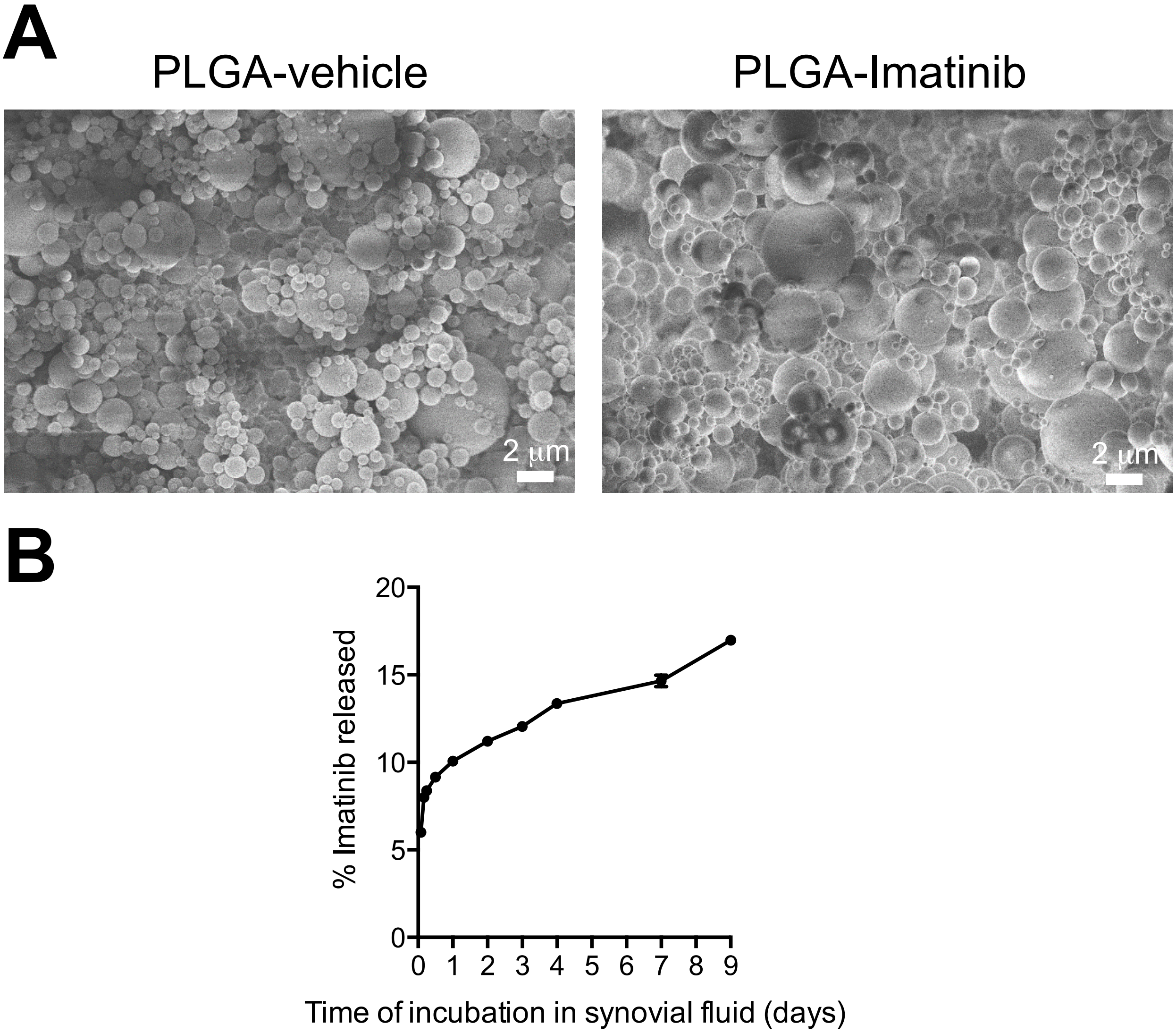


**Supplementary Figure S2.** ***In vitro*** **analysis of imatinib-loaded PLGA nanoparticles. A.** Microscopic picture of vehicle-loaded and imatinib-PLGA nanoparticles, showing that the diameter of the particles was up to 5 μm with an average size < 2 μm. **B.** Release of drug from imatinib-loaded PLGA nanoparticle incubated in human synovial fluid ('normal human synovial fluid' obtained from BioreclamationIVT, USA). 10 mg of imatinib-encapsulated PLGA particles was suspended in 1 mL of synovial fluid in a 1.5 mL microcentrifuge tube. The suspension was incubated at 37°C on an orbital shaker at 120 rpm. At the indicated time points, the particles in the suspension were spun down for 30 seconds using a mini centrifuge (VWR C1413 Galaxy mini centrifuge), and an aliquot of 100 μL was withdrawn for the analysis. The volume was replenished with 100 μL of fresh synovial fluid. The incubation was continued after resuspending the particles. The experiment was performed in triplicate and the results are expressed as the mean ± SD. The released drug was quantified using a 4000 QTRAP HPLC-MS/MS System (AB SCIEX) with Shimadzu Prominence LC system, equipped with system controller CBM-20A, Binary LC-20AD pump, and SIL-20AC autosampler. HPLC was performed using the following specifications: mobile phase: 90% methanol/10% water/0.1% formic acid, column: Dionex, Acclaim 120 C8, 5 μm, 50 x 2.1 mm, flow rate: 0.3 ml/min, and injection volume: 10 μL. The HPLC was directly coupled to an AB SCIEX 4000 QTRAP triple quadrupole mass spectrometer with electrospray ionization. The mass spectrometer was operated in positive multiple reaction monitoring (MRM) mode, ion source: turbo spray, resolution: Q1 unit; Q3 unit.
